# Immune Consequences of Metabolic Syndrome and Its Treatment: A Cytokine Perspective

**DOI:** 10.1111/imm.70166

**Published:** 2026-07-26

**Authors:** Isabela Ghetti Macedo Isaac, Carolina do Prado Servian, Fernanda de Oliveira Feitosa de Castro, João Lucas Lima Calandrini de Azevedo, Ronaldo Moraes Preto, Miriam Leandro Dorta, Daniela Espíndola Antunes, Simone Gonçalves Fonseca

**Affiliations:** ^1^ Instituto de Patologia Tropical e Saúde Pública Universidade Federal de Goiás Goiânia GO Brazil; ^2^ Hospital de Urgências de Goiânia, Secretaria de Estado da Saúde de Goiás Goiânia GO Brazil; ^3^ Pontíficia Universidade Católica de Goiás Goiânia GO Brazil; ^4^ Hospital das Clínicas, Faculdade de Medicina Universidade Federal de Goiás Goiânia GO Brazil; ^5^ Instituto de Investigação Em Imunologia – Instituto Nacional de Ciências e Tecnologia – III‐INCT SP Brazil

**Keywords:** cellular immunity, cytokines, glucose‐lowering medication, immune response, lipid‐lowering medication, metabolic syndrome

## Abstract

Metabolic Syndrome (MetS) is characterized by a set of clinical conditions that elevate cardiovascular risk and may interfere in several pathways of the immune system, as well as the medications used for its treatment. The aim of this study was to evaluate antigen‐specific and polyclonal cellular immune responses in patients with MetS. Thirty‐two participants were divided into three groups: individuals with MetS in use of medication for metabolic conditions (MST), individuals with MetS without treatment (MS), and healthy donors (HD). Blood samples were collected, and peripheral blood mononuclear cells were obtained and stimulated in vitro with peptide pools to evaluate antigen‐specific and polyclonal responses. Supernatants were collected for quantification of interleukin‐2 (IL‐2), interferon‐γ (IFN‐γ), tumor necrosis factor‐α (TNF‐α), and interleukin‐10 (IL‐10). Intragroup comparisons revealed that the MS group showed a more diverse cytokine production compared to the other groups after antigen‐specific stimulation, that could represent a more vigorous immune activation. The comparison of groups revealed that the MST group produced lower levels of IL‐2 and IL‐10 than HD and lower levels of TNF‐α than the MS group after polyclonal stimulation. This attenuated response in the MST group may have multiple causes, such as the inhibitory effects of hyperglycemia on cytokines production and/or the use of medication for metabolic conditions that may exhibit anti‐inflammatory effects. Therefore, the MS group demonstrated a broader cytokine production profile after antigen‐specific stimulation, whereas the MST group exhibited quantitatively lower cytokine production in response to polyclonal stimulation. The implications of these findings require further investigation.

## Graphical abstract

Peripheral blood mononuclear cells (PBMCs) from individuals with metabolic syndrome (MetS) who were not using glucose‐and/or lipid lowering medication (GLLM) exhibited a broader cytokine production profile after in vitro antigen‐specific stimulation than PBMCs from individuals with MetS using such medications and from healthy donors. PBMCs from individuals with MetS who were using GLLM demonstrated quatitatively lower cytokine production after in vitro polyclonal stimulation compared with PBMcs from individuals without GLLM and healthy donors.

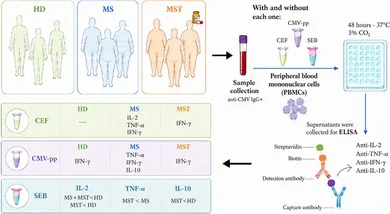

## 1 Introduction

Metabolic Syndrome (MetS) is a heterogeneous medical condition characterized by a group of disorders, which together confer a heightened risk of cardiovascular diseases [1]. It is diagnosed by the presence of three or more of the following conditions: abdominal adiposity, elevated blood glucose and triglycerides levels, hypertension, and decreased serum high‐density lipoproteins (HDL‐c) [2]. The prevalence of this condition has increased in recent years, affecting 20%–30% of the population in the United States [3], Europe [4], China [5] and developing countries [6]. In Brazil, the prevalence of MetS is estimated to approach 30% [7], with a higher incidence among obese individuals and increased with advancing age, especially in women [8].

The MetS is thought to be a state of persistent low‐grade chronic inflammation, with elevated levels of plasma inflammation markers such as interleukin‐6 and C‐reactive protein [9, 10]. Insulin resistance, lipid imbalance, and adipokine dysregulation, which are part of the pathophysiology of MetS, share some pathways with the immune system. This interaction suggests that when one of these systems is altered, dysfunction also occurs in the other [11], which has been demonstrated in some studies [12, 13].

Metabolic disorders such as obesity and diabetes, conditions that can be part of MetS, have been shown to alter immune function and impair defense against pathogens [14, 15]. Some of possible mechanisms are decreased phagocytic activity, increased leukocytes count, change in peripheral CD4^+^/CD8^+^ T cell ratio, elevated Th1 cells and reduction in regulatory T cells (Treg) of adipose tissue and decrease in the number of memory T cells [13]. Obesity and MetS are associated with a higher risk and worse outcomes for some bacterial and viral infections [16, 17, 18]. Decreased immune responses to hepatitis B, tetanus, and influenza vaccines have been described in those with obesity [19, 20, 21]. Additionally, a reduced humoral immune response to COVID‐19 vaccines has been reported in MetS [22, 23]. Besides that, there are studies describing anti‐inflammatory effects of medications used in MetS, such as metformin, statins, and others [24, 25, 26] and these effects in the presence of infectious challenges remain incompletely clarified.

Thus, this study aimed to evaluate and compare the antigen‐specific and polyclonal immune response of peripheral blood mononuclear cells (PBMCs) in individuals with MetS (in subgroups composed of individuals using and not using medication for metabolic conditions) with those from metabolically healthy donors.

## 2 Methods

### 2.1 Ethical Statement

This project was approved by the Research Ethics Committee of Urgency Hospital of Goiás, Goiania, Brazil (Protocol: CAAE‐19015819200000033). All individuals voluntarily agreed to participate and signed the Free and Informed Consent Form (TCLE).

### 2.2 Study Participants, Clinical Evaluation, and Sample Collection

Participants were outpatients recruited from an endocrinology ambulatory care of a tertiary local hospital, including patients, patient's companions, and paramedical staff.

Inclusion criteria included an age between 30 and 65 and positive CMV IgG test. Exclusion criteria were the use of steroids, antibiotics, and vaccines, as well as the presence of infectious disease symptoms (cough, dyspnea, fever, vomiting, diarrhoea, dysuria, and runny nose) within the previous 30 days, the use of immunosuppressive drugs at any time, and a history of autoimmune disease, acute myocardial infarction, stroke, heart, eye and kidney disease, or malignant neoplasm. This information was obtained through a screening questionnaire that also included inquiries regarding their medical history and medication usage.

Individuals were clinically evaluated concerning their weight, height, abdominal circumference, and blood pressure.

Blood samples were collected early in the morning after 12 h fasting period. Fasting plasma glucose (fluoride plasma) and lipid profile (colorimetric enzymatic) quantification were performed. Plasma and serum samples were obtained by centrifugation, aliquoted, and maintained at −80°C until analyses.

MetS was defined by the presence of at least three of the following five risk factors: abdominal obesity (elevated abdominal circumference, population specific definitions), elevated triglycerides (≥ 150 mg/dL), reduced HDL cholesterol (< 40 mg/dL in males and < 50 mg/dL in females), hypertension (systolic blood pressure ≥ 130 or diastolic blood pressure ≥ 85 mmHg) and/or high fasting plasma glucose (> 100 mg/dL) [2].

Individuals were divided into three different groups: Healthy donors (HD), those individuals who met no more than one MetS criteria; Metabolic Syndrome individuals without treatment (MS), those individuals who were positive for 3 or more criteria for MetS and were not taking medication for metabolic condition (glucose—and/or lipid ‐lowering medication); Metabolic Syndrome individuals under pharmacological treatment (MST), those who were positive for 3 or more criteria for MetS and were in use of at least one medication for their metabolic condition (glucose—and/or lipid ‐lowering medication) for a minimun of 1 year.

Additionally, analyses were performed comparing the HD group with the combination of both groups comprised of people living with MetS, with or without use of medication (MS + MST).

The medications that each individual was taking to lower their blood sugar were displayed, as they can be used in various combinations (metformin, sulfonylureas, sodium‐glucose cotransporter‐2 (SGLT‐2) inhibitors, glucagon like peptide‐1 receptor agonists (GLP‐1 RA), dipeptidyl peptidase‐4 (DPP‐4) inhibitors, pioglitazone, and insulin). Each individual's utilization of the lipid‐lowering medication, such as statins (simvastatin, atorvastatin, rosuvastatin, or pitavastatin), or fibrates was also documented.

### 2.3 Cell Preparation

PBMCs were isolated from study participants using Ficoll–Hypaque density gradient centrifugation (density 1.077 g/L, Ficoll‐PaquePlus, GE Healthcare, Uppsala, Sweden). Cell count and viability (> 98% viability) were determined using trypan blue staining (Gibco, Grand Island, USA). The cells were then resuspended in a solution containing fetal bovine serum (FBS) with 10% dimethyl sulfoxide (DMSO) and cryopreserved in liquid nitrogen.

### 2.4 PBMC Stimulation With Peptides

For evaluating the antigen‐specific and polyclonal cellular immune responses in participants, peptide pools derived from Cytomegalovirus pp65 antigen (CMVpp65), Cytomegalovirus, Influenza, and Epstein–Barr (CEF) virus were used, since these viruses commonly cause infections in the general population leading to generation of memory immune responses [27]. *Staphylococcal enterotoxin B* (SEB) was used as a polyclonal stimulus.

PBMCs (4 × 10^6^ cells/ml) from HD, MS and MST were thawed and incubated at 37°C with 5% CO_2_ for 2 h. Cells were then washed and resuspended in Roswell Park Memorial Institute (RPMI) 1640 medium (Lonza, Walkersville, USA) supplemented with 0.1% penicillin, 0.1% streptomycin (GIBCO, Grand Island, USA), and 5% FBS. Cells were cultured in 48‐well plates (Costar, Corning, USA), 0.5 mL per well (2 × 10^6^ cells) and incubated for 48 h at 37°C in a 5% CO2 atmosphere, in 4 different conditions: Cytomegalovirus peptide pool (CMVpp65 peptide set, National Institutes of Health (NIH) AIDS Reagent Program:12014, 8 μg/mL); CEF (Cytomegalovirus, Influenza e Epstein–Barr peptide pool—CEF Control Peptide Pool, NIH AIDS Reagent Program:9808, 2 μg/mL); SEB (Sigma Aldrich, 2 μg/mL); cells without stimulus were included, in order to measure the spontaneous cytokine production. Non‐stimulated control cells were incubated with dimethyl sulfoxide (DMSO, Sigma) at the same concentration as that used to dilute the peptide pools. Supernatants were collected after 48 h of incubation and maintained at −80°C until cytokine quantification assays were performed.

### 2.5 Cytokine Quantification

The concentrations of cytokines (IL‐2, TNF‐α, IFN‐γ, and IL‐10) in the culture supernatants were quantified using enzyme linked immunosorbent assay (ELISA) commercial kits (BD OPTEIA Human‐ BD Biosciences Pharmingen OptEIA, San Diego, CA, USA), according to manufacturer's instructions. Detection limits for each cytokine were: IL‐2 (7.8–500 pg/mL), TNF‐α (7.8–500 pg/mL), IFN‐γ (4.7–300 pg/mL), and IL‐10 (7.8–500 pg/mL).

In addition to the absolute cytokine concentrations obtained under stimulated and non‐stimulated conditions, we also analyzed the relative cytokine production. The relative value was calculated in relation to the non‐stimulated (spontaneous) condition, by subtracting the cytokine level in the non‐stimulated condition from that in the stimulated condition (CMV, CEF, or SEB) for each sample. This approach was chosen to account for individual variability in spontaneous cytokine production and to better reflect the specific response to stimulation.

### 2.6 Immunoenzymatic Assay for Anti‐CMV IgG

Plasma samples from all participants were screened for the presence of IgG antibodies to CMV using a commercial immunoassay kit (CMV IgG; DIA.PRO Diagnostic Bioprobes SRL) according to the manufacturer's instructions. The results were recorded in international units per millilitre (IU/mL). Values greater than 0.5 IU/mL were considered positive for anti‐CMV IgG.

### 2.7 Statistical Analysis

Data were analyzed using SPSS (Windows 21.0—Statistical Package for the Social Sciences; SPSS Inc., Chicago, IL, USA) and Graph Pad Prism software version 8.1.2 (GraphPad Software Inc., San Diego, CA, USA). Kolmogorov–Smirnov and Shapiro–Wilk were used as tests of normality of variable distribution. A *p* value of more than 0.05 in both tests was considered a normal distribution. Chi‐squared and Fisher's exact tests were used to compare differences in absolute and relative data frequencies. Quantitative data were presented as the median and interquartile range. T‐student test (parametric variables) and Mann–Whitney (non‐parametric variables) were used to compare differences among groups. Wilcoxon signed‐rank test (non‐parametric variables) and T‐Student (parametric variables) tests were used for pair‐wise difference analysis. Spearman (non‐parametric variables) and Pearson (parametric variables) correlation tests (*r* and *p*‐values) were used to determine the association between the results. A value of *p* < 0.05 was considered statistically significant.

## 3 Results

### 3.1 Clinical and Laboratorial Characteristics of Individuals

In this study, 32 individuals were included, and they all tested positive for CMV. 27 individuals (84.4%) were female and 5 (15.6%) were male. The individuals were divided into three groups according to clinical and laboratory characteristics, as stated by the inclusion criteria: MST, MS, and HD.

The MST group comprised 10 individuals, all of whom were using metformin. In addition to the utilization of metformin, one individual was also using sulfonylurea, another one insulin, and another one sulfonylurea plus insulin. Furthermore, 5 individuals in the MST group were taking statins (atorvastatin or rosuvastatin). The medications for metabolic conditions used by individuals of the MST group are presented in Table 1.

**TABLE 1:** Medications for metabolic conditions used by individuals of MST group.

MST	Statins (atorvastatin/rosuvastatin)	Metformin	Insulin (NPH)	Sulfonylurea (gliclazide)
1	—	X	—	—
2	X	X	—	—
3	—	X	—	—
4	X	X	—	—
5	—	X	—	—
6	—	X	—	—
7	X	X	—	X
8	X	X	X	—
9	X	X	X	X
10	—	X	—	—

*Note:* X: use of the medication, −: not use of medication. *The table did not include any references to additional glucose and lipid‐lowering medications that were not used by any individual.

The individual's ages ranged from 30 to 63 years. All three groups shared similar demographic characteristics in terms of age, sex, levels of education, smoking habit, alcohol consumption, and physical activity, with no significant statistical differences (Table 2).

**TABLE 2:** Comparison of sociodemographic characteristics among patients in the HD, MS and MST groups.

	HD (*N* = 10)	MS (*N* = 12)	MST (*N* = 10)	*p*
Age, Med (min–max)	43 (33–62)	43 (30–61)	54 (32–63)	0.200^#^
Sex, *N* (%)				
Female	9 (33.3)	9 (33.3)	9 (33.3)	0.750^##^
Male	1 (20.0)	3 (60.0)	1 (20.0)	
Levels of education, *N* (%)				
Primary	0 (0.0)	3 (75.0)	1 (25.0)	0.531^##^
Secondary	5 (33.3)	6 (40.0)	4 (26.7)	
Higher	5 (38.5)	3 (23.1)	5 (38.5)	
Smoking, *N* (%)				
Yes	0 (0.0)	0 (0.0)	0 (0.0)	N/A
No	10 (31.3)	12 (37.5)	10 (31.3)	
Alcohol intake, *N* (%)				
No	7 (28.0)	8 (32.0)	10 (40.0)	0.313^##^
Yes	3 (42.9)	4 (57.1)	0 (0.0)	
Exercise habit, *N* (%)				
Yes	3 (50.0)	0 (0.0)	3 (50.0)	0.125^##^
No	7 (26.9)	12 (46.2)	7 (26.9)	

*Note:* Statistic test: (^#^) ANOVA test (^##^) Fisher–Freeman–Halton test.

Abbreviations: BMI: Body mass index, kg/m2: kilograms/m2; HD: healthy donors; kg (kilograms); Med (min—max): Median (minimum—maximum); MS: Metabolic Syndrome without treatment; MST: Metabolic Syndrome under treatment; *N* (%): absolute frequency (% relative frequency); N/A: not applicable.

There were significant differences in all metabolic issues related to MetS between HD × MS and HD × MST, according to the group definition parameters (weight *p* = 0.001, Body mass index [BMI] *p* < 0.001, number of positive criteria for MetS *p* < 0.001, abdominal circumference [AC] *p* < 0.001, HDL *p* = 0.001, triglycerides [TG] *p* < 0.001, fasting plasma glucose [FGP] *p* = 0.001, systolic blood pressure [SBP] *p* < 0.001, and diastolic blood pressure [DBP] *p* < 0.001; Table 3).

**TABLE 3:** Comparison of clinical and laboratory characteristics among patients in the HD and MS and MST groups.

	HD	MS	MST	*p*
	Med (min–max)	Med (min–max)	Med (min–max)	
Weight (kg)	63.5 (50–77)	85.6 (65–130)	96 (52–120)	0.001^#^*^a,b^
BMI (kg/m^2^)	24 (19–29)	31 (26–45)	38 (23–44)	< 0.001^#^*^a,b^
Number of positive criteria for MetS	0 (0–1)	4 (3–4)	4 (3–5)	< 0.001^##^*^a,b^
AC (cm)	76 (61–99)	104 (94–120)	111 (78–129)	< 0.001^#^*^a,b^
HDL (mg/dl)	61 (36–111)	32 (26–55)	37 (30–50)	0.001^##^* ^a,b^
TG (mg/dl)	66 (36–111)	174 (108–344)	191 (50–352)	< 0.001^##^*^a,b^
FPG (mg/dl)	83 (74–95)	95 (71–296)	126 (88–203)	0.001^##^*^b^
SBP (mmHg)	110 (90–130)	140 (130–150)	125 (110–140)	< 0.001^##^*^a^
DBP (mmHg)	70 (60–84)	90 (80–110)	75 (70–90)	< 0.001^##^*^a,c^

*Note:* Statistic test: (^#^) ANOVA test; (^##^) Kruskal‐Wallis's test. *p < 0.05. Significance is given as: a = control versus MS; b = control versus MST; c = MS versus MST.

Abbreviations: AC: abdominal circumference; BMI: body mass index; cm: centimetre; DBP: diastolic blood pressure; FPG: fasting plasma glucose; HD: healthy donors; HDL: high density lipoproteins; kg/m^2^: kilograms/m^2^; kg: kilograms; Med (min—max): Median (minimum—maximum); MetS: metabolic syndrome; mg/dl: milligram/deciliter; mmHg: millimetres of mercury; MS: Metabolic Syndrome without treatment; MST: Metabolic Syndrome under treatment; SBP: systolic blood pressure; TG: triglycerides.

When comparing the MS + MST with the HD group, there was no significant difference concerning age, but a significant difference related to metabolic criteria (weight *p* < 0.001, BMI *p* < 0.001, number of positive criteria for MetS *p* < 0.001, AC *p* < 0.001, HDL *p* < 0.001, TG *p* < 0.001, FPG = 0.001, SBP *p* < 0.001, and DBP *p* = 0.001; Table 4).

**TABLE 4:** Comparison of clinical and laboratory characteristics between patients in the HD and MS + MST groups.

	HD	MS + MST	*p*
	Med (min—max)	Med (min—max)	
Age (Years)	43 (33–62)	49 (30–63)	0.716^#^
Weight (kg)	63.5 (50–77)	91 (52–130)	< 0.001^#*^
BMI (kg/m^2^)	24 (19–29)	33 (23–45)	< 0.001^#*^
Number of positive criteria for MetS	0 (0–1)	4 (3–5)	< 0.001^##*^
AC (cm)	76 (61–99)	108 (78–129)	< 0.001^#*^
HDL (mg/dl)	61 (36–111)	36 (26–55)	< 0.001^##*^
TG (mg/dl)	66 (36–111)	177 (50–352)	< 0.001^##^
FPG (mg/dl)	83 (74–95)	104 (71–296)	0.001^##*^
SBP (mmHg)	110 (90–130)	130 (110–150)	< 0.001^##*^
DBP (mmHg)	70 (60–84)	80 (70–110)	0.001^##*^

*Note:* Statistic test: (^#^) T de Student test; (^##^) Mann–Whitney test. *p < 0.05.

Abbreviations: AC: abdominal circumference; BMI: Body mass index; cm: centimetre; DBP: diastolic blood pressure; FPG: fasting plasma glucose; HD: healthy donors; HDL: High Density Lipoproteins; kg/m^2^: kilograms/m^2^; kg: kilograms; Med (min—max): Median (minimum—maximum); MetS: metabolic syndrome; mg/dl: milligram/deciliter; mmHg: millimetres of mercury; MS: Metabolic Syndrome without treatment; MST: Metabolic Syndrome under treatment; SBP: systolic blood pressure; TG: triglycerides.

A comparison of the clinical and laboratory characteristics of the MS and MST groups (both with MetS) revealed statistically significant differences between the groups in the number of positive criteria for MetS, which was higher in the group under treatment (*p* = 0.036) and blood pressure measures, which were higher in the untreated group (SBP *p* = 0.07, DBP *p* = 0.088) (Table 5).

**TABLE 5:** Comparison of clinical and laboratory characteristics between individuals in the MS and MST groups.

	MS	MST	*p*
	Med (min–max)	Med (min–max)	
Age	43 (30–61)	54 (32–63)	0.086^#^
Weight	85.6 (65–130)	96 (52–120)	0.752^#^
BMI	31 (26–45)	38 (23–44)	0.253^#^
Number of positive criteria for MetS (0–5)	4 (3–4)	4 (3–5)	0.036^##*^
AC (cm)	104 (94–120)	111 (78–129)	0.373^#^
HDL (mg/dl)	32 (26–55)	37 (30–50)	0.346^##^
TG (mg/dl)	174 (108–344)	191 (50–352)	0.722^##^
FPG (mg/dl)	95 (71–296)	126 (88–203)	0.093^##^
SBP (mmHg)	140 (130–150)	125 (110–140)	0.007^##*^
DPB (mmHg)	90 (80–110)	75 (70–90)	0.002^##*^
HbA1C	5.6 (4.9–14.0)	6.7 (5.2–10.3)	0.088^##^

*Note:* Statistic test: (^#^) ANOVA test; (^##^) Mann–Whitney test; * *p* < 0.05.

Abbreviations: AC: abdominal circumference; BMI: Body mass index; cm: centimetre; DBP: diastolic blood pressure; DBP: diastolic blood pressure; FPG: fasting plasma glucose; HbA1C: glycated hemoglobin; HDL: high density lipoproteins; kg/m2: kilograms/m2; kg: kilograms; Med (min–max): Median (minimum—maximum); MetS: metabolic syndrome; mg/dl: milligrams/deciliter; mmHg: millimetres of Mercury; MS: Metabolic Syndrome without treatment; MST: Metabolic Syndrome under treatment; SBP: systolic blood pressure; SBP: systolic blood pressure; TG: triglycerides.

### 3.2 Intragroup Analysis of Cytokine Production Following Antigenic Stimulation

To assess the individual immune responses to stimuli by antigenic peptides from CMV and CEF, and polyclonal stimulation with SEB, a pairwise analysis was conducted between the absolute values of the condition without any stimulus (NS), that is, spontaneous production, compared to the absolute values of stimulated production levels.

The IL‐2 production assessment did not demonstrate a statistically significant difference between the spontaneous values and the values obtained after the CMV stimulus in the HD, MS, and MST groups. Only MS exhibited a significantly increased production of IL‐2 following the CEF peptide pool stimulus (Figure 1A,B). However, for the production of TNF‐α, there was a statistically significant difference for the MS group between spontaneous production and the production after CMV (*p* < 0.01) and CEF (p < 0.01) stimuli (Figure1D,1E). All groups showed significantly elevated levels of IL‐2 (HD *p* < 0,01, MS *p* < 0.001 and MST *p* < 0,01) and TNF‐α production (HD *p* < 0,01, MS *p* < 0.01 and MST *p* < 0,01) after stimulation with the superantigen SEB, compared to their respective spontaneous production (Figure 1C,F).

**FIGURE 1:**
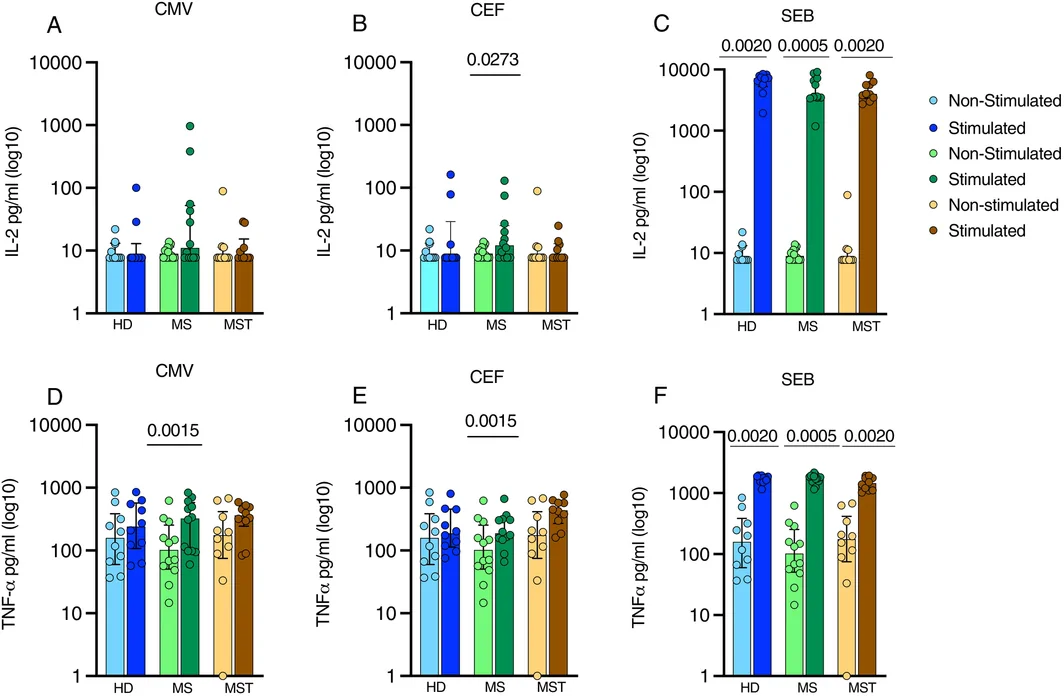
Production of cytokines IL‐2 and TNF‐α after stimulation with CMV and CEF peptide pools, and SEB. Paired analyses before and after stimulus. The values expressed in pg/ml correspond to the spontaneous production and production of cytokines following stimulation of CMV and CEF peptide pools, and SEB by PBMC (2 × 10^6^ cells/well) of the respective individuals of each study group, determined by ELISA after 48 h. CEF: Cytomegalovirus, Epstein Barr e Influenza peptide pool; CMV: Cytomegalovirus peptide pool; HD: Healthy donors; IL‐2: Interleukin 2; MS: Metabolic Syndrome without treatment; MST: Metabolic Syndrome under treatment; NS: No stimulus (spontaneous production); SEB: Staphylococcal enterotoxin B; TNF‐α: Tumor necrosis factor‐alpha. Bars represent the median, vertical lines indicate the interquartile range, and dots represent individual cytokine values. Statistical test: Wilcoxon signed‐rank test. Statistical significance considered when *p* < 0.05.

The production of IL‐10 was shown to be increased with statistical significance after the stimulation of PBMC with CMV peptide pool (*p* < 0.01) and SEB (*p* < 0.001) compared to non‐stimulated cells for patients with untreated Metabolic Syndrome (Figure 2A,C). The CEF stimulus did not significantly impact production of IL‐10 in any of the groups (Figures 2B). With the exception of HD after CEF, all groups experienced a statistically significant increase in IFN‐γ production following the application of all stimuli (CMV stimulus HD *p* < 0.01, MS *p* < 0.01 and MST *p* < 0.01; CEF stimulus MS *p* < 0.01 and MST *p* < 0.01; SEB stimulus HD *p* < 0.01, MS *p* < 0.01 and MST *p* < 0.01) (Figure 2D–F).

**FIGURE 2:**
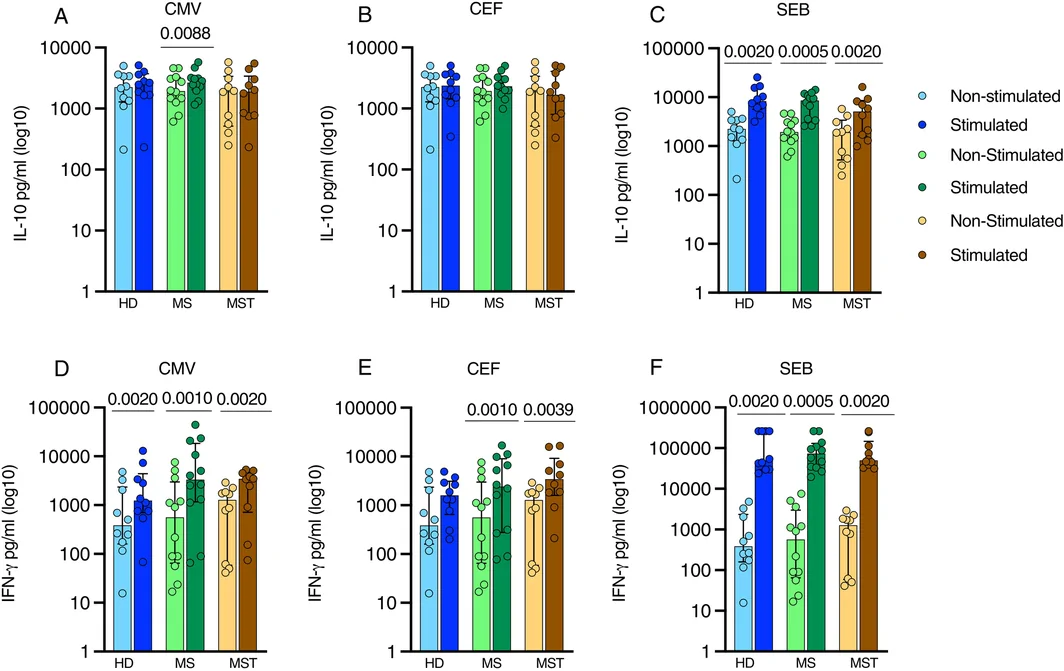
Production of cytokines IL‐10 and IFN‐γ after stimulation with CMV and CEF peptide pools, and SEB. Paired analyses were performed considering cytokine production before and after stimulus. The values expressed in pg/mL correspond to the spontaneous production (non‐stimulated) and stimulated cytokine production by PBMC (4 × 10^6^ cells/ml) of the respective individuals of each study group, determined by ELISA from individuals in each study group, determined by ELISA after 48 h. CEF: Cytomegalovirus, Epstein Barr and Influenza peptide pool; CMV: Cytomegalovirus peptide pool; HD: Healthy donors; IFN‐γ: Interferon gamma; IL‐10: Interleukin 10; MS: Metabolic Syndrome without treatment; MST: Metabolic Syndrome under treatment; NS: No stimulus (spontaneous production); SEB: *Staphylococcal enterotoxin* B. Bars represent the median, vertical lines indicate the interquartile range, and dots represent individual values. Statistical test: Wilcoxon signed‐rank test. Statistical significance considered when *p* < 0.05.

Collectively, all groups demonstrated a significant elevation in the secretion of all analyzed cytokines (IL‐2, TNF‐α, IL‐10, and IFN‐γ) following SEB stimulation (Figures 1C,F and 2C,F).

### 3.3 Comparison of Cytokine Production Among Groups Following Antigenic Stimulation

The cytokine production after stimulation with CMV and CEF peptide pools, and SEB, was evaluated by discounting spontaneous production and comparing the results between groups. For IL‐2, there was no difference in production between the groups after stimulation with CMV and CEF peptide pools (Figure 3A,B).

**FIGURE 3:**
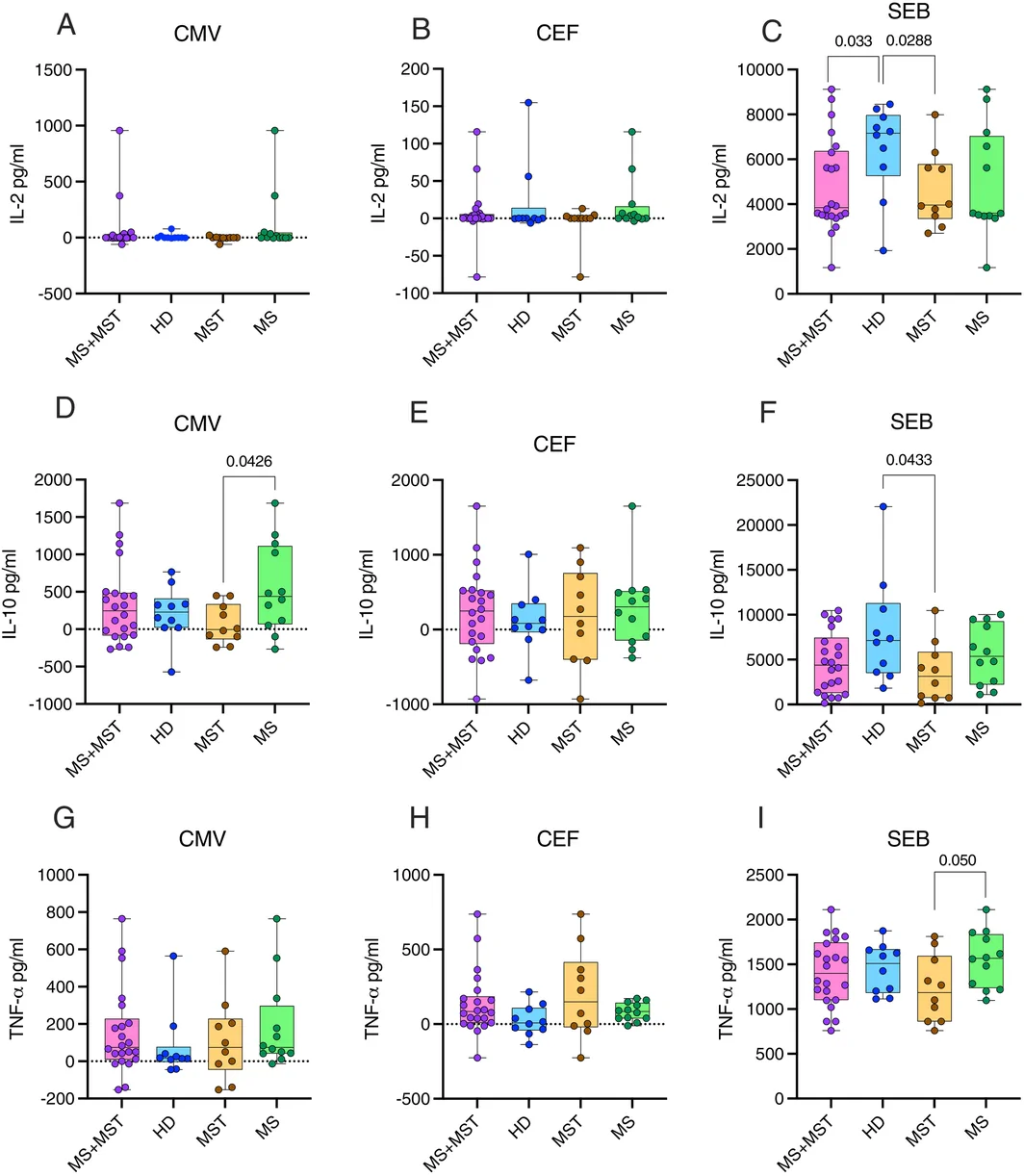
Comparison of relative cytokine production among groups. Production after stimulus minus the value of spontaneous production of IL‐2 (A, B, C), IL‐10 (D, E, F), TNF‐α (G, H, I) after the stimuli with CMV (A, D, G), CEF (B, E, H) and SEB (C, F, I). All groups were compared to each other. CEF: Pool of cytomegalovirus, Epstein–Barr and Influenza peptides; CMV: Cytomegalovirus peptide pool; HD: Healthy donors; IL‐10: Interleukin 10; IL‐2: Interleukin 2; MS: Metabolic Syndrome without hypoglycemic and/or lipid lowering treatment group; MST: Metabolic Syndrome with glucose or/and lipid lowering treatment group; SEB: Staphylococcal enterotoxin‐ B; TNF‐α: Tumor necrosis factor‐alpha. Boxplots show median (center line), interquartile range (box), and whiskers indicating minimum and maximum values. Each symbol represents the cytokine production of each individual. Values represent cytokine production after background subtraction (stimulated minus non‐stimulated condition); negative values were retained and plotted as obtained. Statistical significance was considered when *p* < 0.05 and was done comparing 2 by 2 condition using Mann–Whitney test for non‐parametric variables.

Conversely, after the SEB stimulus, there was a significantly higher production of IL‐2 by the HD group with a median of 7159.2 pg/mL (range 1932.4–8457.7), in comparison to MS + MST groups, which had a median of 3849.7 pg/mL (range 1166.9–9123.4; *p* = 0.033), and in comparison to the MST group, which had a median of 3960.7 pg/mL (range 2702.5–7987.6; *p* = 0.0288) (Figure 3C).

It was found that the result of IL‐10 production following CMV stimulation was significantly lower in the MST group, median of −4.2 pg/mL (−242.0–448.0), than in the MS group, median of 438.1 pg/mL (range −266.0–1686.3; *p* = 0.0426) (Figure 3D). There was no difference between the groups for the stimulus with CEF (Figure 3B,E,H). After SEB stimulation, the MST group had a lower median of IL‐10 production, 3130 pg/mL (range 156.0–10468.0) compared to the HD group, median of 7132.3 pg/mL (range 1827.8–22042.0; *p* = 0.0433) (Figure 3F).

The production of TNF‐α was similar between the groups after stimulation with CMV and CEF (Figure 3G,H). In contrast, following SEB stimulation, there was a trend toward a difference between the MS group, median of 1567.4 pg/mL (range 1097.4–2110.8) and the MST group, median of 1184.4 pg/mL (range 758.7–1810.9; *p* = 0.050) (Figure 3I).

There were no differences between the groups in the production of IFN‐γ against any of the stimuli (data not shown).

### 3.4 Correlation Analysis

The correlation analysis for the MS group, between the variables showed negative associations between FPG and IL‐10 levels (SEB), AC and IL‐10 (CEF), and a positive correlation between HDL and IL‐10 (CEF) (Figure 4A).

**FIGURE 4:**
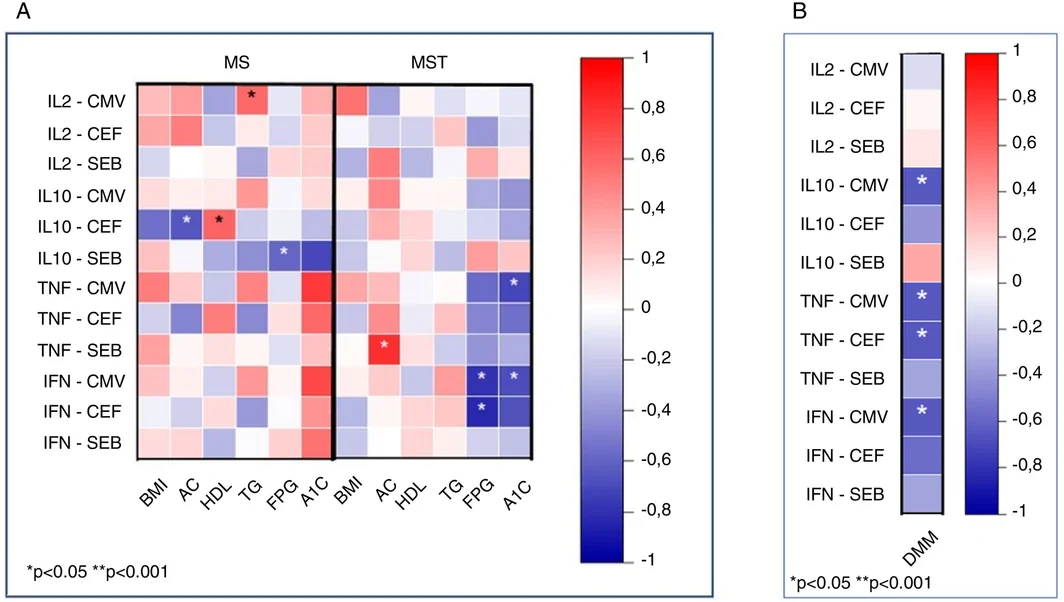
(A) Analysis of the correlation between clinical and laboratory characteristics of individuals in the MS and MST groups and their cytokine production in response to CMV, CEF and SEB stimuli. (B) Analysis of the correlation between the number of glucose‐lowering drugs used in the MST group and their cytokine production in response to CMV, CEF and SEB stimuli. A1C: Glycated hemoglobin; BMI: Body mass index, AC: Abdominal circumference; DMM: diabetes medication; HDL: High density cholesterol; IgG‐CMV: Titles of IgG immunoglobulin for cytomegalovirus; IL‐10: Interleukin 10; IL‐2: Interleukin 2; MS: Metabolic Syndrome without use medication for metabolic conditions group; MST: Metabolic Syndrome under medication for metabolic conditions group; TG: Triglycerides, FPG: Fasting plasma glucose; TNF‐α: Tumour necrosis factor‐alpha. Pearson and Spearman correlation tests.

In the MST group, there was a positive correlation between AC and TNF‐α production (SEB). Negative correlations were identified between FPG and IFN‐γ (CMV and CEF), glycated hemoglobin (A1C) and IFN‐γ (CMV), and A1C and TNF‐α (CMV) (Figure 4A).

There was also a negative correlation between the number of glucose‐lowering agents used and the production of IFN‐γ (CMV), TNF‐α (CEF and CMV), and IL‐10 (CMV) in the MST group (Figure 4B).

## 4 Discussion

Metabolic syndrome is significantly prevalent worldwide [28], and it has been shown to have a detrimental effect on the outcomes of viral, bacterial, and protozoan infectious diseases and on the efficacy of vaccinations in specific cases [29, 30, 31, 32]. Characterizing cytokine production in this population in response to antigen‐specific and polyclonal stimuli may help elucidate the immunological mechanisms underlying the differences observed in clinical outcomes. To the best of our knowledge, there are no previous data addressing this analysis.

The present study revealed minor variances in the pattern of cytokine production by PBMCs among individuals with metabolic syndrome and healthy donors. Nonetheless, further disparities emerged upon analyzing the group of individuals with metabolic syndrome separately into those users and non‐users of medication for metabolic conditions.

There was a broader variety of cytokines produced by PBMCs of MS after an antigen‐specific stimulus, and a quantitative limitation of cytokine production by PBMC of MST after stimulus, especially a polyclonal one.

A significant increase in antigen‐specific stimulated production of TNF‐α and IL‐10 was observed in the MS group, which did not occur in the other groups. This difference suggests a more diverse immune response within the MS group, since HD and MST responded significantly only with IFN‐γ production. It is important to emphasize that the MS group presented a different behavior from the HD, which would be the standard reference parameter in the experiment. Previous studies described higher levels of TNF‐α production in individuals with MetS or related conditions, exhibiting a more inflammatory serum cytokine profile and PBMCs responsiveness compared to controls [33, 34]. In the present study, this occurred together with the production of IL‐10, which has a regulatory profile. Despite the significant increase in IL‐10 production in the MS group, no difference was observed in the ratio between pro‐inflammatory cytokines and IL‐10 (data not shown).

A negative association was observed between AC and IL‐10 production (CEF), while a positive association between HDL and IL‐10 production (CEF) was found in the MS group. In the MST group, a positive correlation was detected between AC and TNF‐α production induced by SEB. Such correlations, between unfavourable metabolic parameters and elevated levels of pro‐inflammatory cytokines and decreased levels of anti‐inflammatory plasma cytokine production, have been previously established [35, 36, 37]. This association was demonstrated in antigen‐ and polyclonal‐stimulated PBMCs cytokine production, in an unprecedented manner, in this study. In individuals with abdominal obesity, an adverse pattern of adipokines is part of the events that characterize adipose tissue dysfunction, with elevated leptin and decreased adiponectin, which may correlate with increased pro‐inflammatory immune activity [38, 39]. The typical obese adipokine profile could be an explanation for the associations found between metabolic parameters and cytokine production; however, those dosages were not performed.

Conversely, the present study was unable to establish any correlation between BMI and any cytokine production. Potentially as a result of the samples' dimensions. Literature has shown that the data regarding the production of regulatory cytokines in MetS has been contentious. Though pro‐inflammatory cytokine production has been consistently described [33, 40], an increase in both Th1 and Th2 cytokines has also been observed in individuals with MetS [41, 42]. In general, obesity impairs Th2 responses while enhancing type 1 inflammatory responses [43]. Regulatory cells are the predominant population in lean adipose tissue and maintain tissue homeostasis through type 2 cytokines [44]. Thus, Th2 responses are the most prevalent in lean individuals and offer protection against insulin resistance [45].

Nevertheless, this phenomenon is not universally present, particularly out of the adipose tissue. A homeostatic trend toward the Th2 phenotype was observed in the peripheral blood of the morbidly obese [46]. It has been described that leptin, which is elevated in obesity, enhanced the Th2 response and promoted allergic airway disease [47].

When considered collectively, the evidence suggests that the immune landscape in metabolic syndrome and obesity is not a straightforward dichotomy between Th1 and Th2. Rather, it seems that metabolic syndrome induces a systemic immune activation that is characterized by an increase in both Th1 and Th2 cytokines.

This study found lower levels of IL‐2 in comparison to other cytokines for all groups, potentially due to the timing of sample collection relative to the peak production of this cytokine in cell cultures [48]. Notably, there was a significant difference in the production of IL‐2 after SEB stimulus between the group with MetS (MST + MS) and the HD, in which the MetS groups had lower levels detected. While previous reports have indicated increased IL‐2 expression in the adipose tissue of obese individuals, correlating with inflammatory markers, fasting blood glucose, and glycated hemoglobin [49], the present analysis was conducted on stimulated peripheral blood mononuclear cell cultures rather than adipose tissue. It appears that the lower IL‐2 production occurred at the expense of the treatment group, since it remains significant in the comparison between the HD and the MST groups.

The present study found that the IL‐2 production was significantly stronger after the CEF stimulus and not for CMV (Figure 1) and that, on the other hand, the production of IL‐10 was significantly higher in the CMV stimulus and not for CEF when analyzing the antigen‐specific response (Figure 2). In accordance with these findings, it has been described that PBMCs stimulated with CMV pool produce very low IL‐2 in comparison to IFN‐γ [50] and it is probably a consequence of the terminally differentiated effector memory phenotype of the dominant CMV‐specific cell population [51]. In the CEF peptide pool, the production of IL‐2 tends to be slightly more expressive than in the CMV peptide pool due to the stimulus of the EBV and influenza effector memory cells [52].

The CEF peptide pool is designed to bind MHC class I molecules and activate CD8^+^ T cells exclusively [53]. So, the production of IL‐10 is expected to be low since CD4+ T cells are the dominant source of antigen‐specific IL‐10 production [54].

The attenuated cytokine (IL‐2, IL‐10, and TNF‐α) response of the MST group after a polyclonal stimulus may be attributed to three mechanisms: the potential anti‐inflammatory action of the medication used for metabolic conditions, the more significant glucose alterations, and greater metabolic syndrome abnormalities detected in this group, as discussed below.

The anti‐inflammatory actions of drugs for metabolic conditions could have directly resulted in the reduction of cytokine production observed. In MST, the potential contribution of the use of medication in the attenuated cytokine production in the MST group was suggested through the correlation analysis, which indicated a negative correlation between the quantity of glucose‐lowering agents administered and the production of IFN‐γ (CMV), TNF‐α (CEF and CMV), and IL‐10 (CMV). It has been shown before that glucose‐lowering and lipid‐lowering medications may have a direct inhibitory action on the immune response through diverse mechanisms, some of them listed below [24, 25, 26, 55, 56, 57, 58]. In the present study, all the individuals in the MST were on metformin, five were taking statins, two individuals were taking sulfonylurea, and two were under insulin. The anti‐inflammatory effects of metformin have been explained by the activation of adenosine monophosphate‐activated protein kinase (AMPK), which leads to cytokine‐induced NF‐ⱪƁ inhibition [59], by the blockade of the phosphoinositide‐3‐kinase‐protein‐kinase B pathway [60] and through the induction of phosphatase and tensin homologue, which is an inflammatory response negative regulator that affects cell survival, growth, and proliferation [55]. Statins can increase the expression of cytokine secretion suppressor 3 and 7 in macrophages, decrease the expression of NF‐ⱪƁ [61, 62], and negatively affect proteins involved in the presentation and processing of antigens, such as Rab and Rho, which generate an accumulation of inactive forms and inhibition of these signalling molecules [63, 64]. Statins have also been related to inhibition of the proliferative T cell response [65]. It has been demonstrated that sulfonylurea inhibits the NLR NOD‐, LRR‐, and pyrin domain‐containing protein 3 (NLRP3) inflammasome [66, 67] and reduces lymphocyte proliferation upon stimulation [57]. All these medications have been related to a reduction in pro‐inflammatory cytokine production [68, 69, 70, 71]. Metformin has been associated with an increase in anti‐inflammatory cytokine production [24, 70, 72] and statins have been linked to either increases or decreases in IL‐10 expression, depending on the context of the disease and the cells involved [73, 74]. Therefore, these medications' anti‐inflammatory properties may account for MST's decreased response in terms of cytokine production following SEB. Regarding insulin, it is well known that its receptor is important for the immunostimulation of T cells and that insulin resistance in T cells contributes to immune dysfunction in vitro [75]. However, the effect of exogenous insulin on T cells requires further investigation.

The immune system may also be affected by other medications used to treat metabolic conditions that were not used by any of the participants in this study. The presumed mechanisms of anti‐inflammatory action of GLP‐1 RA are the inhibition of NF‐κB and IkB degradation induced by TNF‐α and the activation of JNK [76, 77]. DPP‐4 inhibitors may affect the immune response by modulating CD26, a membrane‐anchored form of DPP‐4 that functions as a co‐stimulatory molecule and is involved in the activation of T cells [78, 79]. Pioglitazone is a synthetic ligand with high affinity for peroxisome proliferator‐activated receptors gamma (PPAR‐γ) [80] whose induction is required for alternative activation of macrophages in generating anti‐inflammatory phenotypes for adipose tissue [81]. The anti‐inflammatory properties of the SGLT‐2 inhibitors are associated with a decrease in NLRP3 activation and IL‐1 β production [82, 83], a reduction in the production of superoxides by leukocytes [84], and in the expression of TLR4 in renal tissue [85]. Fibrates activate the PPARα receptors, which can disrupt the immune system by suppressing proinflammatory transcription factors like NF‐κB, AP‐1, and STAT [86].

The benefits of the pharmacological treatment concerning metabolic control reduction in morbidity and mortality are undeniable and highly expressive. Conversely, the immune system's reactions to these medications remain inadequately understood. The modulation of the immune system may be intriguing in certain circumstances, as in cases of severe inflammation, such as sepsis, where the utilization of statins, metformin, and DPP‐4 inhibitors was associated with decreased mortality rates [87, 88, 89]. However, it may not be as desirable in situations where the complete immune response is essential, such as vaccines. Some studies have suggested that the efficacy of influenza vaccines may be diminished by the use of statins, while others have either refuted these findings or suggested that statin use may be associated with an increased risk of influenza, regardless of vaccination status [90, 91]. Van Niekerk et al. (2024) [92] have addressed the potential negative effects of GLP‐1 RA on vaccine efficacy in a recent revision; still, there is currently no concrete evidence to support this assertion.

In the present study, the contribution of hyperglycemia to the decreased cytokine production in response to stimulation was demonstrated by the negative correlations detected in the analysis. Specifically, for the MS group, there was a negative correlation between FPG and IL‐10 concentrations when cells were stimulated with polyclonal agent (SEB). Similarly, in the MST group, negative correlations were detected between FPG and IFN‐γ (CMV and CEF), glycated hemoglobin and IFN‐γ (CMV), and glycated hemoglobin and TNF‐α (CMV). It has been described that hyperglycemia has inhibitory effects on the function of immune cells, including lymphocytes [93]. An animal‐based study demonstrated that chronic exposure to hyperglycemia led to impairment in T lymphocyte function, characterized by reduction in the proliferative response after stimulation, an increase in the rate of oxygen consumption, and elevated basal glycolytic activity [94]. Also, a study by Richard et al. (2017) compared cytokine production by PBMCs in patients with obesity, without metabolic changes in comparison to patients with obesity and diabetes. They found that there was a reduction in the response of IL‐2 production to polyclonal stimulation with phytohemagglutinin in the group with obesity and diabetes [95].

In addition to blood glucose and glycated hemoglobin, in the treatment group the individuals presented a greater number of positive criteria for MetS than those of the untreated group, which could reflect greater intensity of the syndrome in this group.

It is imperative to underscore that the group that was receiving treatment still met the metabolic syndrome diagnosis despite taking medication, in accordance with the group's inclusion criteria, since even in treatment, most individuals do not achieve remission.

A 2026 randomized trial discovered that metabolic syndrome remission was achieved by only approximately 28% of participants at 24 months, despite the intensive lifestyle intervention [96]. Globally, the proportion of individuals receiving diabetes treatment with optimal glycemic concentrations was approximately 41.6%, and in some regions it can be even lower [97]. It is significantly more difficult to regulate all abnormal metabolic components simultaneously than to regulate individual risk factors [98]. In 2015–2018, only about 21% of United States' adults with diabetes achieved all three targets simultaneously: glycemic control (HbA1c < 7%), blood pressure (< 140/90 mmHg), and cholesterol (non‐HDL < 130 mg/dL) [99].

Metabolic syndrome is often described as a condition of low‐grade chronic inflammation, characterized by constant and low production of acute phase proteins and cytokines (such as C‐reactive protein, IL‐6, TNF‐α, IL‐1β) [10]. The most attenuated cytokine production response observed in MST when exposed to the polyclonal stimulus could be due to exposure to a chronically inflammatory state, potentially leading to an exhausted‐like cellular phenotype [100]. It is possible that elevated basal cytokine levels lead to the induction of a state of tolerance, which would result in decreased cytokine production after stimulation [101]. However, in the present study, phenotypic analyses of cells were not performed to characterize cellular exhaustion.

Additionally, the more severe metabolic issues observed in the MST could be attributable to the duration of the illness. As the majority of metabolic alterations are asymptomatic and detected as laboratory findings, it was not feasible to ascertain the duration of Mets for all individuals. For the MST, it was possible to estimate the length of treatment, which varied from 1 to 20 years, with a median of 5 years.

It has been demonstrated that long‐term exposure to metabolic disturbances was associated with an increased risk of complications, including cardiovascular diseases [102]. The presence of heart, eye, and kidney disease was an exclusion criterion in this study. However, the likelihood of the group undergoing treatment experiencing a more prolonged illness is substantial. Although there is a dearth of supporting evidence in the literature, it is possible to infer that problems related to compromised immune response may be more serious in longer‐term metabolic disease. The current study identified a negative correlation between the duration of the treatment and the production of IFN‐γ after CMV stimulation (*R* = −0.6689 and *p* = 0.0394); however, the correlation was not statistically significant for IL‐2, IL‐10, and TNF‐α (Supporting Information).

Due to the study design, it was not feasible to determine whether the greater severity of hyperglycemia and greater intensity of MetS in individuals undergoing treatment, the utilization of medications with anti‐inflammatory action by individuals in this group, or even the association between these factors were the root causes of the observed disparities. Irrespective of the exact cause, these results emphasize that this group responded in a different pattern in both antigen‐specific and polyclonal stimulus.

In order to ensure that the cytokine production values were not influenced by confounding factors, it was imperative to establish groups with comparable metabolic parameters. This was accomplished in this study since there were no statistically significant differences in metabolic parameters between MS and MST. Even though the metabolic parameters of the two subgroups of patients with MetS (MS and MST) did not differ statistically, the median values for MST were above the normal range, while the median values for MS were within the normal range in the analysis of glycated hemoglobin and fasting blood glucose. The same occurred in the quantity of positive criteria for MetS. MST exhibited more positive criteria, albeit the median was identical and no significant statistical difference was found. It is conceivable that future research could more accurately demonstrate the impact of pharmacological treatment on cytokine production by contrasting groups with metabolic parameters that are even more similar, both with and without treatment.

Larger cohorts may have revealed more substantial differences, which represent a limitation of this study. Although the number of male participants in the present study was low, it occurred evenly in all the groups, without any statistical disparities among them. This was, however, another limitation of this study.

Patients with metabolic syndrome constitute a heterogeneous population, as their diagnosis is based on the presence of three out of five defined criteria. The diverse metabolic parameters that comprise the group may affect the immune response in different ways. However, the objective of this study was to evaluate patients with metabolic syndrome, rather than to separately evaluate their distinct characteristics, which has been the focus of previous research [103, 104]. Comparing groups with precise metabolic alterations would not be feasible in this study given the large number of individuals needed. Nevertheless, correlation analysis was conducted considering each individual metabolic parameter.

Additional studies should clarify the impact of those in vitro immunological responses in a real‐life scenario. Investigating the impact of MetS associated with the use of related drugs on individuals' immune response while facing an infectious disease could support the potential necessity of developing specific vaccination and treatment protocols for these individuals.

## 5 Conclusions

Collectively, the data indicate that the group with MetS who were not under medication for metabolic conditions exhibited a more extensive range of cytokine (IL‐2, IL‐10, and TNF‐α) production in response to an antigen‐specific stimulus than healthy donors and those with MetS who were receiving treatment. Additionally, the cytokine production response after polyclonal stimulation was quantitatively lower for patients with MetS using metabolic medication than those without treatment (TNF‐α) and the healthy controls (IL‐2 and IL‐10). The implications of these responses require further investigation.

## Author Contributions

**Isabela Ghetti Macedo Isaac:** conceptualization, methodology, investigation, formal analysis, data curation, writing – original draft, visualization. **Carolina do Prado Servian:** formal analysis, investigation, writing – review and editing. **Fernanda de Oliveira Feitosa de Castro:** formal analysis, investigation, writing – review and editing. **João Lucas Lima Calandrini de Azevedo:** investigation, formal analysis, writing – review and editing. **Ronaldo Moraes Preto:** investigation, writing – review and editing. **Miriam Leandro Dorta:** supervision, writing – review and editing. **Daniela Espíndola Antunes:** supervision, writing – review and editing. **Simone Gonçalves Fonseca:** conceptualization, methodology, investigation, resources, formal analysis, validation, data curation, project administration, funding acquisition, writing – review and editing.

## Funding

This work was supported, in part, by the *Fundação de Amparo à Pesquisa do Estado de Goiás* (FAPEG), Brazil (Grant No. 201810267001508) and the Conselho Nacional de Desenvolvimento Científico e Tecnológico (CNPq), Brazil (Grand Nos. 486715/2012‐4 and 313198/2022‐5) and the Coordenação de Aperfeiçoamento de Pessoal de Nível Superior (CAPES), Brazil.

## Conflicts of Interest

The authors declare no conflicts of interest.

## Supporting information


**Figure S1:** Correlation between years of treatment and cytokine production to CMV, CEF, and polyclonal (SEB) stimulation. Cytokine production (expressed as the difference between stimulated and non‐stimulated cells) in cell culture supernatants was correlated with the years of treatment in patients with metabolic syndrome (MST). The Spearman correlation test was applied, and *r* and *p* values are shown. A *p*‐value < 0.05 was considered significant.

## Data Availability

The data that support the findings of this study are available from the corresponding author upon reasonable request.
